# Biomimetic materials based on zwitterionic polymers toward human-friendly medical devices

**DOI:** 10.1080/14686996.2022.2119883

**Published:** 2022-09-13

**Authors:** Kazuhiko Ishihara

**Affiliations:** Division of Materials and Manufacturing Science, Graduate School of Engineering, Osaka University, Osaka, Japan

**Keywords:** Biomaterials, zwitterionic polymers, phosphorylcholine group, surface modification, antifouling property, lubrication, implantable artificial organs, contact lenses

## Abstract

This review summarizes recent research on the design of polymer material systems based on biomimetic concepts and reports on the medical devices that implement these systems. Biomolecules such as proteins, nucleic acids, and phospholipids, present in living organisms, play important roles in biological activities. These molecules are characterized by heterogenic nature with hydrophilicity and hydrophobicity, and a balance of positive and negative charges, which provide unique reaction fields, interfaces, and functionality. Incorporating these molecules into artificial systems is expected to advance material science considerably. This approach to material design is exceptionally practical for medical devices that are in contact with living organisms. Here, it is focused on zwitterionic polymers with intramolecularly balanced charges and introduce examples of their applications in medical devices. Their unique properties make these polymers potential surface modification materials to enhance the performance and safety of conventional medical devices. This review discusses these devices; moreover, new surface technologies have been summarized for developing human-friendly medical devices using zwitterionic polymers in the cardiovascular, cerebrovascular, orthopedic, and ophthalmology fields.

## GRAPHICAL ABSTRACT

**Figure:**
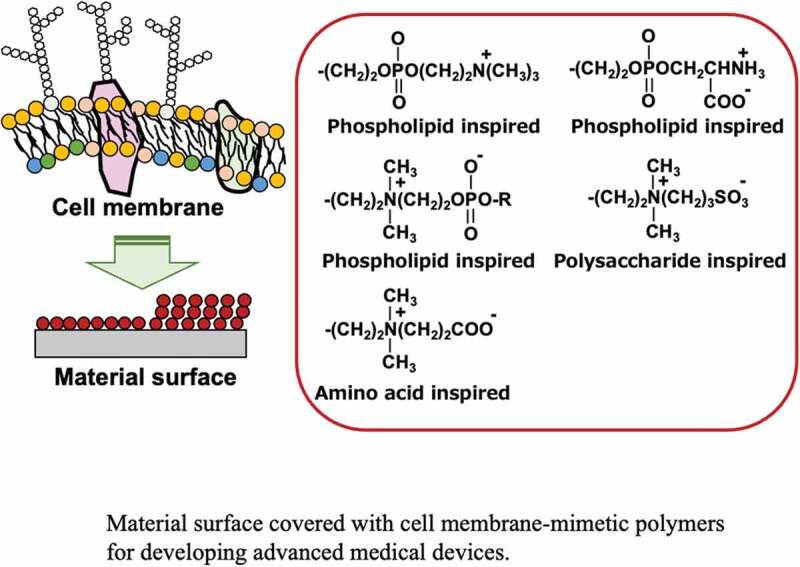

## 1. Introduction

Medical devices are indispensable for treatment and diagnosis in current healthcare. Artificial organs that replace some of the functions of the living system and diagnostic test kits that capture and diagnose molecules present in the body have made significant contributions to medical care to sustain human life and improve quality of life. They have been developed based on materials science and bioengineering [1]. However, the large gap between living systems and artifacts can result in medical devices causing disorders in living organisms [2–4]. Physicians and patients must weigh the effects of the treatment against its impact on the organism and make choices regarding its use. To evaluate the effects, it is essential to create an interface that mediates the relationship between a medical device and living tissues [5,6]. Living tissues are embryologically divided by cells to produce the necessary properties and functions, forming unique shapes and features to suit the individual [7]. Additionally, these tissues can precisely express their role in a fluid-mediated environment and have specific mechanical properties. Furthermore, natural tissue can be processed without causing damage to the living system when it is no longer needed, and can partially be regenerated [8]. These features are challenging to imitate with artificial material systems that are used to fabricate medical devices. In other words, in medical devices, which are formed by combining multiple parts made from industrially standardized and straightforward materials, there is concern that loss of function will not be repaired and the device will further damage living systems [9]. The fabrication of materials that appropriately mediate the interface between different biological tissues and medical devices is a central theme in biomaterials research [10].

In living systems, cells are highly organized to maintain their biological activity. Cells produce energy through molecular reactions and are responsible for accurate transmission and control of information. If the information transfer fails, biological activities may cease [11]. For suitable molecular recognition, the reactivity with the target molecules must be enhanced, while unwanted molecular reactions must be prevented. Characteristic properties of the cell surface play an essential role in this function [12]. The concept of improving the performance of materials and equipment by mimicking the superior properties of biological systems is scientifically termed ‘biomimetics’ [13,14]. Illustrative examples include morphological mimicry of water-repellent lotus leaves and molecular mimicry of energy production, as in the molecular mechanism of photosynthesis. This review focuses on polymer molecular design methods based on functional mechanisms relating to antifouling and lubricious interfaces, using biomimetics at the molecular level.

The interface between living and artificial systems often induces disturbances owing to their very different properties [15–17]. For example, biological fouling by marine organisms such as shellfish and algae that adhere to boats, fishing nets, and window surfaces of giant aquarium tanks is a phenomenon commonly observed in ecosystems [18,19]. This can lead to the complete loss of functions of the observation equipment, sensors, and other devices, making it impossible to respond in an emergency. The removal of these contaminants requires a significant amount of time and money. In the atmosphere, the contamination of materials and paints reduces the energetic efficiency of solar panels and wind power generation equipment and induces secondary problems such as freezing [20]. Such phenomena occur on common material surfaces, and chemicals and paints that leak metal ions have been used to prevent them. However, these metal ions induce environmental pollution.

**Figure 1.**
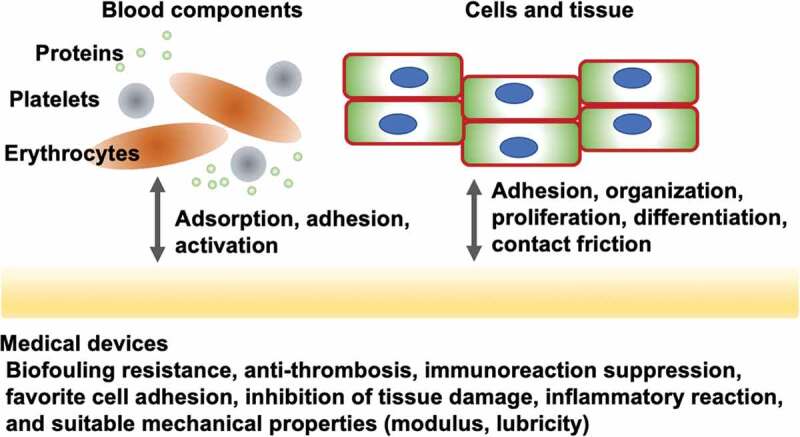
Representative biological reactions of medical devices and their required surface properties.

Various biological responses and infections may impair medical devices used in vivo; this may reduce the effectiveness of treatment and increase infected tissue, which can endanger lives [21–24]. These results are based on a series of biological reactions that are strongly related to various biomolecules and cellular systems present in living systems. Figure 1 shows representative biological reactions at the surface of materials. When medical devices are used to treat patients, they invade the living body. Living organisms undergo a healing process to repair this invasion and maintain life [25]. A change in the equilibrium state (homeostasis) is known as a disease. Homeostasis is a dynamic state in which the coordination of biomolecules occurs in a time-dependent manner. If the material in contact with the dynamic state of the biological tissue is static or its properties are very different from those of the biological tissue, a new biological reaction occurs that eliminates the two without applying them to each other.

Here, the focus is on the polymers produced by biomimetic concepts that result in biomaterials due to the unique structure and properties of living organisms. The development and characteristics of medical devices using these polymers have also been discussed.

**Figure 2.**
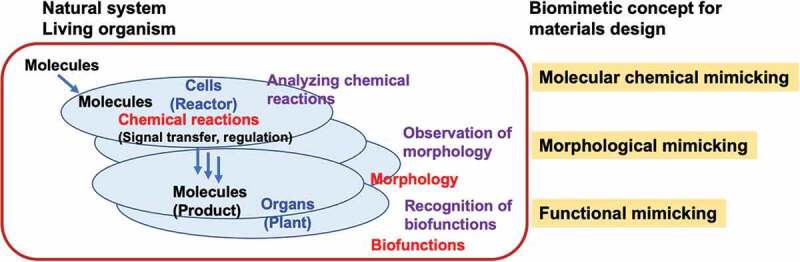
Biomimetic concepts for materials design.

Recently, the validity of biomimetic concepts has been confirmed in various fields, contributing to society [26,27]. Figure 2 shows a schematic representation of the biomimetic concept. Biomimetics can be classified into three types according to their levels: morphological mimetics that are based on the morphology of living organisms, molecular chemical mimetics based on the structure of biomolecules, and biofunctional mimetics that artificially reproduce the necessary elements by elucidating the functions expressed by living organisms at the molecular level. Development of low aerodynamic drag inspired by the form of a bird’s bill and the form of a ship that mimics the shape of a fish are examples of morphological mimicry. Examples of biomimicry at the molecular level include the synthesis of polyesters, polyamides, and polypeptides that mimic the scientific structures and formation processes of proteins and nucleic acids. In contrast, mimicking biological functions requires an understanding of the root causes of functional expression. The key is to achieve a complex system in which many biomolecules form sophisticated reaction systems and structures using a simple artificial system structure. Furthermore, simplifying this process is essential for implementing functional biomimicry in devices.

**Figure 3.**
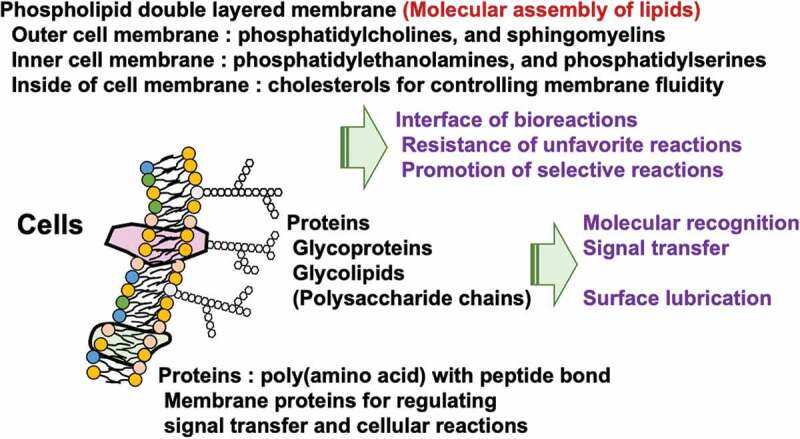
Biological components and their characteristics of the cell membrane surface.

For the development of biomimetic polymers, attention has been paid to the polar groups of phospholipid molecules present on the surfaces of cell membranes [28–30]. Figure 3 shows a schematic representation of the cell membrane structure, including various biomolecules and their functions. The cell membrane basically takes a phospholipid bilayer membrane structure; the polar groups of the phospholipid molecules that form this bilayer membrane are asymmetric [31]. Most phospholipid molecules inside the cell membrane have weakly acidic phosphatidylethanolamine or acidic phosphatidylserine as polar groups. These regulate the ion balance in the cell membrane and are responsible for the transmission of information. In contrast, phosphatidylcholine and sphingomyelin, which have a phosphorylcholine group with a neutral charge state, occupy most of the surface in contact with the extracellular aqueous phase. Glycoproteins and membrane proteins are present on the cell membrane surface, accurately capturing signal molecules from the outside and transmitting information into the cell. Currently, the non-specific capture of information molecules on the cell membrane surface causes a significant decrease in cell function and additionally generates an unfavorable biological reaction as a secondary stimulus. The normal outer membrane itself has a structure that suppresses non-specific reactions with biological components, and it is considered that the phosphorylcholine group plays a role at the functional group level [32,33]. Therefore, biomimetic polymers, in which phospholipid molecules are introduced into polymers, have been studied [34–36]. It has been found that the introduction of these polymers to surfaces of medical devices can prevent biological contamination and inhibit the reaction of biological tissues, thus extending the life of medical devices [37–41].

## 2. Biomimetic polymers with zwitterionic polar groups

**Figure 4.**
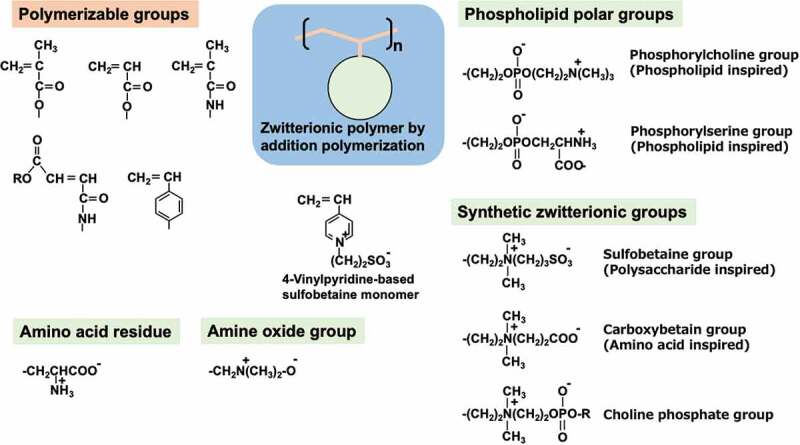
Molecular design of zwitterionic monomers.

Biomacromolecules play a significant role in biological reactions and regulation in living organisms. They are produced mainly by amino acid derivatives or phosphoric acid derivatives through polycondensation. The composition, structure, and sequence of each unit determine the primary structure, and intramolecular interactions give a specific morphology of the whole molecules. In terms of the chemical structure of these biomacromolecules, amino acids have carboxyl and amino groups, and phospholipid molecules have phosphate and ammonium groups. Therefore, both anions and cations exist in the molecules; these structures are termed ‘zwitterionic’ structures. In recent years, zwitterionic polymers have become the subject of considerable research in synthetic polymers, based on a molecular design inspired by the structure of biomacromolecules. Their synthesis, physical properties, and functional evaluations have been extensively studied [42–44]. In particular, their ability to generate novel polymers through addition polymerization is highly advantageous in research and subsequent practical applications because of the stability of the molecules and ease of molecular design when adding functions. Therefore, the creation of monomers with functional properties is indispensable. A variety of monomers have been synthesized with various changes in the structures of polymerizable and zwitterionic groups for use as raw materials for zwitterionic polymers [45–47]. The typical chemical structures of monomer are shown in Figure 4. Combinations of various polymerizable and zwitterionic groups that can undergo addition polymerization are shown. Also monomers have been synthesized to produce polymers via polycondensation, polyaddition, or ring-opening polymerization, and there is a growing variety of these compounds. The most studied monomer is a compound with a zwitterionic phosphorylcholine group attached to its side chain [48]. As shown in Figure 4, the phosphorylcholine group has a phosphate anion and trimethylammonium cation, both of which are well-balanced in the molecule, therefore the overall charge is neutral.

**Figure 5.**
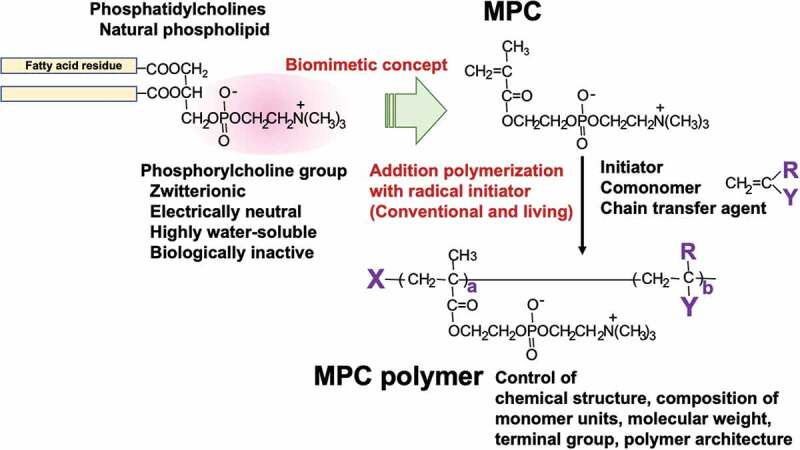
Chemical structure of biomimetic MPC polymers.

In 1987, Ishihara *et al*. developed efficient procedures for the synthesis and purification of 2-methacryloyloxyethyl phosphorylcholine (MPC), a methacrylate monomer with a phosphorylcholine group [49]. Figure 5 shows the molecular design and chemical structure of MPC and its polymer. Based on this process, the Japanese chemical company NOF Co. has been producing high-purity MPC since 1999. Currently, MPC is readily available as a reagent to researchers worldwide, resulting in considerable advances in the field. MPC can easily be polymerized by a normal radical reaction and copolymerization with other vinyl monomers [50,51]. Therefore, polymers with various properties can be obtained by changing the chemical structure and composition of the monomer unit and controlling the molecular weight. Furthermore, as recent living radical polymerization methods can be applied, polymers having a precisely defined molecular structure, including block-type and graft-type copolymers, have been synthesized [52–56]. The most representative MPC polymer for surface coating on substrates is poly(MPC-*co-n*-butyl methacrylate (BMA)), which is random polymer with a 30 mol% MPC unit composition (PMB) [48,49,57].

**Figure 6.**
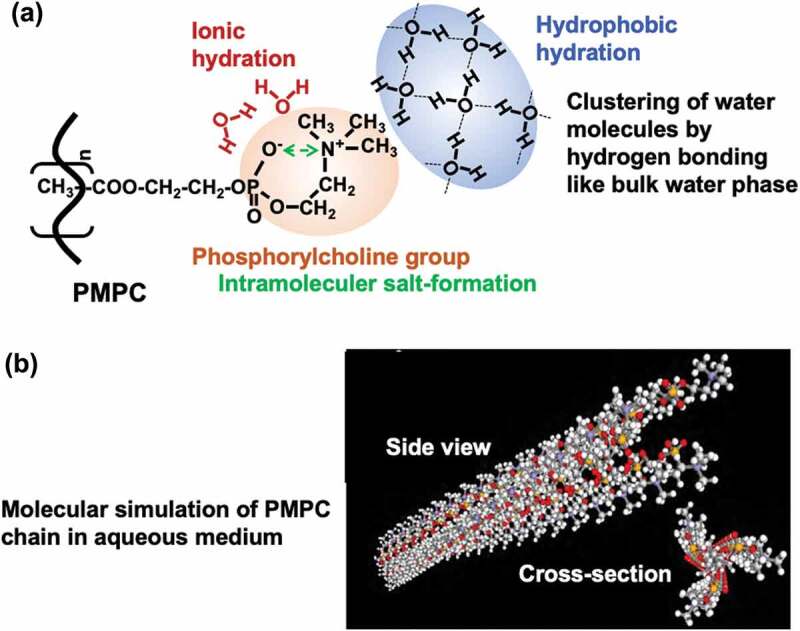
(a) Schematic representation of hydration state of PMPC and (b) molecular simulation image of PMPC chain in aqueous medium.

The hydration state of zwitterionic polymers provides an essential insight into their unique functions and has been abundantly researched [58,59]. The phosphorylcholine group of MPC polymers exhibits a characteristic hydration state in aqueous mediums [60]. Figure 6 shows the hydration structure around the phosphorylcholine group of the assumed poly(MPC) (PMPC) (Figure 6(a)) and the results of the simulation of the molecular structure in water (Figure 6(b)). The PMPC takes rod-like molecular morphology in an aqueous medium and a cross-section of a molecular chain seems fully covered by the main chain with bulky phosphorylcholine groups. The number of hydrogen bonds in the surrounding water molecules was analyzed by Raman spectroscopy of aqueous solutions of the PMPC, and it was found that the number of hydrogen bond defects per MPC unit was approximately −0.1 [61]. This is significantly smaller than the 1.0 value found for poly(ethylene glycol) (PEG), a representative non-electrolyte-based water-soluble polymer, and the 4.5 value for poly(acrylic acid) (PAA), a polyelectrolyte. The dissolution of water-soluble polymers generally requires the hydration of water molecules into the polymer chain; therefore, hydration affects the structure of the surrounding water molecules. However, in the case of PMPC, hydration is considered to occur with little or no effect. As described above, the chemical structure of the phosphorylcholine group suggests the possibility of hydrophobic hydration, rather than strong hydrogen bonding or ionic hydration [29,62]. This is explained by the clustering of water molecules by hydrogen bonding with each other. As the MPC unit is electrically neutral, the phosphate anion and trimethylammonium cation are close together, resulting in an outward orientation of the three methyl groups attached to the nitrogen atom. This provides a suitable site for hydrophobic hydration. It is energetically advantageous for water molecules to adopt a clustered structure around hydrophobic functional groups as they hydrogen bond to other water molecules instead of interacting with the hydrophobic functional groups. Therefore, it can be inferred that the MPC unit has a minimum number of hydrating water molecules around the charged groups. In contrast, water molecules take on a cluster structure equivalent to bulk water, owing to hydrophobic hydration in the surrounding the phosphorylcholine groups. It is reported that the state of water molecules at the phosphatidylcholine-aligned interface is similar to that of the bulk water phase. The mobility of water molecules is not affected by the interface, as measured by two-dimensional heterodyne-detected vibrational sum-frequency generation (2D HD-VSFG) spectroscopy [63]. Molecular dynamics calculations also indicate that the NH_4_+ ions of phosphatidylethanolamine cleave hydrogen bonds between water molecules in the second hydration layer. However, the N(CH_3_)_4_+ ion of phosphatidylcholine has been reported to weakly bind to water molecules in both the primary and secondary hydration layers, increasing the hydrogen bonds between water molecules [64]. Recently, precise thermal analysis has been performed using aqueous zwitterionic polymer solutions, and the relationship between the water structure and polymer structure was reported. According to this, the PMPC aqueous solution contains more anti-freeze and intermediate water than the aqueous solutions containing carboxybetaine polymer or sulfobetaine polymer [65].

PMPC does not undergo significant changes in molecular morphology or solubility with pH or ionic strength in aqueous solutions [66]. This feature differs significantly from that of common polyelectrolytes [67]. Specifically, in polyelectrolyte solutions, a rapid increase in viscosity is generally observed when the polymer concentration is lowered. This is because the counterions bind to the charged functional group and become electrically neutral, creating an electrostatic shielding effect, leaving the functional group when the concentration is low and increasing the effective charge of the polymer chain. As a result, the molecular chain spreads due to electrostatic repulsion. However, because phosphorylcholine groups do not have counterions, the polymer state can only partially depend on the polymer concentration. From the neutron scattering results of PMPC-grafted interfaces, Takahara *et al*. deduced that PMPC chains spread in water and that their state is independent of changes in ionic strength [68]. The *in vivo* environment does not change significantly; therefore, analysis of the surface using the *in vivo* environment is required. MPC and several representative MPC polymers, including PMB, are commercially available as reagents, and their functions are being studied worldwide [48]. In addition, many researchers have synthesized vinyl monomers with phosphorylcholine groups on the side chain and reported their polymerization and polymer solubility (see Figure 4) [69].

In response to research results on polymers with phosphorylcholine groups, there have been many studies on varying zwitterionic polymers and their medical applications [70–78]. These are sulfobetaine and carboxybetaine groups, which are derived from biomolecules such as sulfated polysaccharides and amino acids, respectively. With both positive and negative charges in a single molecule, these polymers are thought to exhibit properties equivalent to those of phosphorylcholine groups; however, they exhibit different characteristics depending on the type of functional group and structure of the substituent. For example, sulfobetaine compounds have a very low water solubility [79,80]. This may be due to the intramolecular sulfonate anion and trimethylammonium cation acting individually, resulting in strong intermolecular electrostatic interactions. The solubility of sulfobetaine compounds in aqueous NaCl solutions is high, and the electrostatic shielding effect of counter ions is remarkable. In other words, sulfobetaine-based polymers show good aqueous solubility under high ionic strength, such as in biological environments. The formation of ionic bonds within and between sulfobetaine groups has attracted attention [81]. The effects of salt precipitation and deposition by multivalent ions in living organisms may be considered for future research when these sulfobetaine compounds are used as biomaterials in biological environments for extended periods. Carboxybetaine compounds have a high water solubility, and this feature is maintained in polymers. When considered in the same way as phosphorylcholine groups, the zwitterionic ionic structure of weak acids and strong bases maintains the ion balance within the molecule, resulting in a good hydration state [82]. Additional zwitterionic groups, such as phosphorylserine [83–85], choline phosphate [65,66,86,87], and amine-oxide [88], can be introduced into the polymer chain, and their fundamental functions are reported in Figure 4. These polymers have unique functions depending on their chemical structures. Additionally, charge-balanced polymer systems based on the equilibrium combination of polyanions and polycations (polyampholytes) have been investigated [89–92]. The application of polymerization and copolymerization of the corresponding vinyl monomers is also advantageous because the chemical structure, composition, and molecular weight of the monomer units can be easily controlled, allowing for the complexation of functions. Advanced living radical polymerization methods have been applied to precisely define polymer structures, making it possible to guarantee safety and other factors [90].

## 3. Zwitterionic polymers at the interface of biomaterials

The interfacial properties required of materials to control reactions with biomolecules and cells can be determined by assuming that the material is a water medium that is exposed to a dynamic environment [93,94]. Interfaces that do not allow cells to adhere must either not adsorb adhesion proteins using the cell adhesion mechanism or not reveal peptide sequences that serve as adhesion ligands through conformational changes in the adsorbed proteins. There have been many reports on the effects of zwitterionic polymers on these interfacial properties [35,69,95,96]. Whitesides et al. examined the protein adsorption behavior of self-assembled membranes with various functional groups and provided molecular insights into the chemical structure of the surfaces that prevent protein adsorption [97]. They have proposed four requirements for inhibition of protein adsorption, these are hydrophilicity, charge neutrality, the presence of hydrogen-bonding acceptor groups, and the absence of hydrogen-bonding donor groups. Phosphorylcholine and carboxybetaine groups satisfy these conditions. Although the carboxybetaine group is pH-dependent, it is electrically neutral under biological conditions. These facts indicate the potential use of zwitterionic polymers as biomaterials.

Compared to PEG-immobilized surfaces, which are generally considered to be able to inhibit protein adsorption, the polymer structure enables stable surface treatment with functional groups. These groups have an affinity to the surface and simple surface treatment processes [98–100], exploiting the fact that the monomer unit expresses the function and thus has a wider range of applications. PEG is a polyether and is therefore easily oxidized [101,102]. Therefore, although PEG is widely used to inhibit protein adsorption at the research level, there are few examples of its application in medical devices for clinical use.

**Figure 7.**
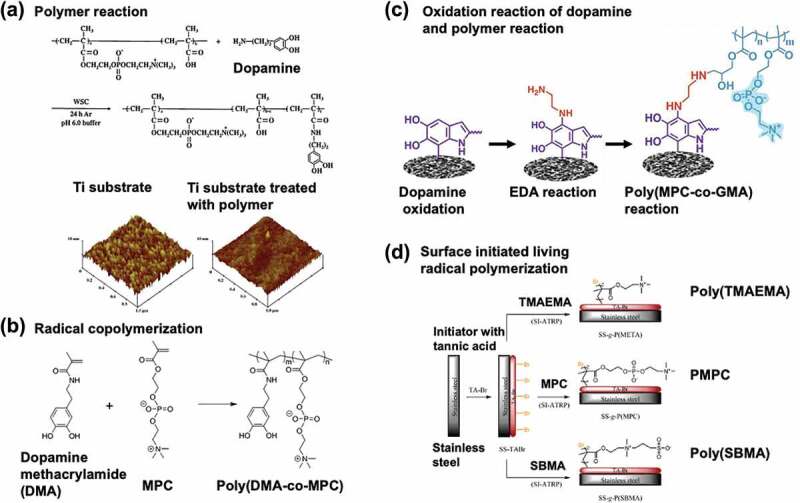
Biomimetic surface immobilization procedure with zwitterionic polymers. (a) MPC polymer with catechol moiety prepared by polymer reaction and the atom force micrographic images at the surfaces titanium substrate before and after surface treatment. Reprinted with permission from [103]. Copyright (2011) elsevier. (b) Copolymerization of MPC with dopamine methacrylamide (DMA). Reprinted with permission from [104]. Copyright (2019) American chemical society. (c) Coating procedure on the substrate by polymer reaction with MPC polymer with the epoxy group following dopamine polymerization. Reprinted with permission from [105]. Copyright (2022) elsevier. (d) Surface-initiated atom transfer radical polymerization (SI-ATRP) after immobilization of the initiator by dopamine chemistry. Reprinted with permission from [106]. Copyright (2015) American chemical society.

When zwitterionic polymers are used to modify material surfaces, it is necessary to bind them to the surface stably using a simple process. These methods have varied applications, depending on the surface modification process and nature of the surface to be modified. Recently, biomimetic processes have attracted considerable attention as shown in Figure 7 [107]. The corresponding reactions revealed that the molecular chemical mechanism of mussel adhesion to rocks mimics that of the chemical structure of the dopamine groups. Therefore, by introducing aromatic polyhydric-hydroxyl groups into the polymer chain, stable polymer-modified layers can be formed. This is done via hydrogen bonding and chelate formation through oxidation reactions between polyhydric hydroxyl-substituted aromatic substituents, which introduces surface crosslinking via the hydroxyl group. This method can be applied to several base materials and may simplify the surface treatment process in cases where not only polymers but also metals and ceramics are used, such as in medical devices [108,109]. It will become an effective surface treatment method in the future when more detailed studies have been conducted on its stability and safety. The binding strength of polyhydric-hydroxyl-substituted aromatic compounds to a wetted surface depends on the number of hydroxyl groups attached to the aromatic ring. To apply this biomimetic process as a surface treatment for zwitterionic polymers, the introduction of polyhydric-hydroxyl-substituted aromatic functional groups has been investigated [107,109,110]. The simplest method is the condensation of dopamine into a zwitterionic polymer with carboxyl groups (Figure 7(a)) [103]. MPC polymers synthesized using this method have been found to reduce cell and bacterial adhesion to metals, such as titanium and silicon substrates [111]. The molecular structure cannot be defined because of the difficulty of 100% substitution in polymeric reactions.

Copolymerization with polyhydric-hydroxyl-substituted aromatic monomers has recently been studied (Figure 7(b)) [104]. Because polyhydric-hydroxyl-substituted aromatic compounds have large radical chain transfer constants, they exhibit poor addition polymerization ability and do not increase in molecular weight. However, this has been overcome using solvents and initiators. Living polymerization techniques can also be applied, and polymers with well-defined structures have been successfully synthesized [112,113]. There are also methods to create more stable and highly functional surfaces, such as introducing reactive polymers to dopamine by forming an oxidative polymerization film on the base material (Figure 7(c)) [105], or graft polymerization by introducing an initiator for living radical polymerization (Figure 7(d)) [106]. These have potential as methods for modifying the surfaces of medical devices, and further research is expected.

As a surface treatment for metals and ceramics, the stable chemical fixation of polymer chains by silane coupling reactions is also effective in manufacturing medical devices. Because this reaction is industrially established and monomers with silane coupling groups are readily available, many studies have been reported [114,115]. MPC polymers with silane coupling groups are synthesized to improve the resistance of protein adsorption and lubricity of the substrates [116,117]. Silane coupling groups and hydroxyl groups are introduced into the polymer chains for the surface treatment of stainless-steel substrates, and intermolecular cross-linking reactions are performed [118]. In both cases, stable surfaces can be obtained near the biological environment.

In addition to the formation of a stable surface-modified layer, chemical durability in a sterilization environment is an essential factor when applied to medical devices. Ethylene oxide gas, high-pressure steam, and radiation sterilizations are currently used, and the effects of these conditions on zwitterionic polymers have been reported [119]. Although the stability of the surface-modified layer on the substrate varies, and the results do not represent the effect of sterilization conditions alone, it has been reported that the thickness of the surface-modified layer on sulfobetaine polymers changes less than 5% after sterilization using an electron beam and approximately 30% when hydrogen peroxide is used. The hydrolysis of ester bonds in the side chains of a representative sulfobetaine polymer, 2-methacryloyloxyethyl)-dimethyl-(3-sulfopropyl)ammonium hydroxide (SBMA) polymer, when autoclaved, is reported to be improved by alternating it with an amide bond [120]. To resolve this problem, poly(4-vinylpyridine)-based sulfobetaine polymers have been synthesized (Figure 4) [121]. However, γ-ray radiation sterilization is mainly used for MPC polymers when used in actual medical devices.

## 4. Medical application of zwitterionic polymers

Research is underway to improve the blood and tissue compatibility of various medical devices using zwitterionic polymers molecularly designed based on biomimetic concepts. Some of these medical devices have been approved by organizations and are used clinically to improve the quality of life of patients. The results are summarized in Table 1 [75,122–136], and several examples of these studies are described in this section.

**Table 1.** List of medical devices using biomimetic zwitterionic polymers.

Zwitterionic group	Polymers	Functions	Surface modification mode	Medical devices	Ref.
Phosphorylcholine	PMPC	Lubricity	Grafting on cross-linked UHMWPE	Artificial hip joint *Approved*	[122]
	Poly(MPC-co-BMA) (PMB)	Blood compatibility	Solution casting on titanium	Implantable blood pump (LVAD) *Approved*	[123]
		Anti-infection	Solution casting on poly(glycolic acid) fibers	Suture *Approved*	[124]
		Protein adsorption resistance	Solution casting on polyurethane	Implantable glucose sensor for wearable artificial pancreas *Approved*	[125]
		Anti-infection Tissue compatibility	Solution casting on PDMS	*Central venous catheter* *Approved*	[126]
	Poly(MPC-co-DMA) (PMD)	Blood compatibility	Solution casting on polypropylene porous membrane	Oxygenator (Artificial lung) *Approved*	[127]
	Poly(MPC-co-DMA-HPMA-co-TMSPMA)	Blood compatibility	Solution casting and reacting on stainless steel wire	Cardiovascular stent; polymer coating stent *Approved*	[128]
		Blood compatibility Drug reservoir	Solution casting and reacting on stainless steel wire	Cardiovascular stent; drug eluting stent *Approved*	[129]
		Blood compatibility Lubricity	Solution casting and reacting on titanium wire	Flow diverter device *Approved*	[130]
	Poly(MPC-co-HEMA-co-EGDMA)	Protein adsorption resistance	Hydrogel formation	Soft contact lens (Omafilcon A) *Approved*	[131]
	Poly(MPC-co-AEMA)	Anti-biofouling Lubricity Tissue compatibility Wetting property	Reaction to silicone hydrogel with carboxy group	Silicone hydrogel contact lens (Lehfilcon A) *Approved*	[132]
Sulfobetaine	Poly(SBMA-co-GMA)	Anti-biofouling	Cross-linking reaction with ethylene diamine on polypropylene fiber	Blood separation device *Approved*	[133]
	PSBMA	Cell adhesion control for preventing posterior capsular opacification	Graft polymerization on initiator-immobilized PMMA surface	Intraocular lens *Under research*	[134]
		Lubricity	Photoinduced Graft polymerization on PDMS	Cochlear implant *Under research*	[75]
Carboxybetaine	Poly(CBAAm-co-BPAAm)	Anti-biofouling Prevention of elution of plasticizer	Solution casting on plasticized PVC tubing	Blood circuit *Under research*	[135]
	Poly(CBMA-co-SMA)	Blood compatibility	Solution casting on polypropylene hollow fibers	Oxygenator *Under research*	[136]

Abbreviations: PMPC: poly(2-methacryloyloxyethyl phosphorylcholine), MPC: 2-methacryloyloxyethyl phosphorylcholine, BMA: *n*-butyl methacrylate, DMA: *n*-dodecyl methacrylate, HPMA: 2-hydroxypropyl methacrylate, TMSPMA: 3-trimetoxysilylpropyl methacrylate, EGDMA: ethylene glycol dimethacrylate, AEMA: 2-aminoethyl methacrylate, SBMA: 2-(methacryloyloxy)ethyl)-dimethyl-(3-sulfopropyl)-ammonium hydroxide, GMA: glycidyl methacrylate, PSMBA: poly((2-methacryloyloxy)ethyl-dimethyl-(3-sulfopropyl)ammonium hydroxide), CBAAm: 1-carboxy-*N,N*-dimethyl-*N*-(3′-acrylamidopropyl) ethanaminium inner salt, BPAAm: *N*-(4-benzoylphenyl) acrylamide, SMA: *n*-stearyl methacrylate.

### 4.1. Medical devices in contact with blood

#### 4.1.1. Cardiovascular devices

Most cardiovascular medical devices are used in an environment in which they are in long-term contact with blood. Therefore, it is important to prevent blood coagulation, which is suppressed through the combined use of anticoagulant therapy. A coronary stent is a medical device that utilizes metal strands to patent a narrowed portion of a blood vessel [137]. A coronary stent is an endovascular treatment that is placed at a predetermined site by a catheter attached to a guidewire from the blood vessel of the thigh. When first developed, the metal surface of the strands was exposed (metal-bearing stent). However, with advances in technology, over the years, the surface was gradually coated with polymers (polymer-coated stent), and now, the polymer layer is even incorporated with drugs (drug-eluting stent). The polymer coating layer acts as a reservoir for drugs that prevent thrombus formation immediately after attachment and the thickening of blood vessels.

**Figure 8.**
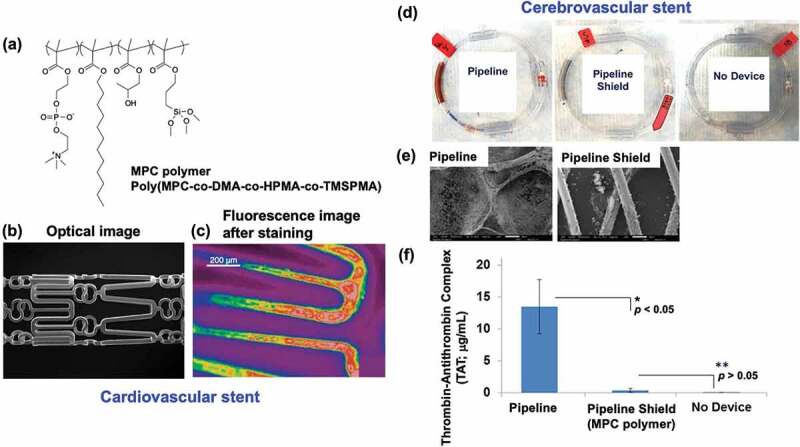
(a) Chemical structure of cross-linkable MPC polymer. (b) Morphology of cardiovascular stent treated with the MPC polymer and (c) fluorescence image of it after staining with rhodamine 6G. Reprinted with permission from [140]. Copyright (2005) Wiley. (d) in vitro blood coagulation test of a cerebrovascular stent using blood circuit method and (e) SEM image of the stent without and with MPC polymer treatment after 60-min contact with whole blood. (f) the concentration of thrombin-antithrombin complex (TAT) in blood after 60-min contact with whole blood. (d), (e), (f) are reprinted with permission from [141]. Copyright (2005) Wiley.

Since the 1990s, coronary stents coated with MPC polymer (BiodivYsio stents, Biocompatibles Ltd.) have been developed and clinically applied [128,137–139]. This is the first example of a long-term *in vivo* implantable medical device that uses an MPC polymer. MPC polymers are quaternary copolymers of MPC, hydrophobic *n*-dodecyl methacrylate (DMA), reactive 3-trimethoxysilylpropylryl methacrylate (TMSPMA), and 2-hydroxypropylryl methacrylate (HPMA), which react directly with the metal oxide layer on the metal surface or between polymer chains through the TMSPMA units [140]. The chemical structure of the MPC polymer (Figure 8(a)), shape, and coating state of the MPC polymer on the stainless-steel stent (Figures 8(b,c)) are seen in Figure 8. The monomer unit composition ratio of the polymer MPC/DMA/TMSPMA/HPMA was 23/47/25/5. An extremely stable polymer coating layer was formed owing to the reaction described earlier. BiodivYsio stents are safe and effective primary devices for the treatment of native coronary artery lesions. No thrombotic events in small-diameter blood vessels have been reported and good clinical results have been obtained [142]. On the surface coated with MPC polymer, 91% of the surface was covered with endothelial cells 5 days after implantation. After 30 days, the surface was completely transferred from the endothelial cell layer to the smooth muscle cell tissue, and the inner surface of the blood vessels was normal. It was confirmed that the stent was stably present in the vascular tissue even after 6 months. Cell adhesion is unlikely to occur on the MPC polymer surface. Therefore, it is suggested that the process of covering the BiodivYsio stent with endothelial tissue must be done such that the strands pass through the endothelial cell layer and slip into the tissue, due to the tension of the stent.

Based on this stent, a new-type Endeavor stent and Endeavor Sprint stent were developed by Medtronic [143]. These drug-eluting stents can sustain the release of zotarolimus, which has an inhibitory effect on cell proliferation. Zotarolimus has antiproliferative and anti-inflammatory effects, and the zotarolimus-sustained-release stent significantly reduced the angiographic restenosis rate compared with the metal stent. The MPC polymer used is a cross-linkable polymer on a metal stent surface. And drug loading is carried out during the coating procedure. In the case of the Endeavor stent, the amount of zotarolimus loaded was controlled at 10 mg/mm-stent length. The MPC polymer layer acts as both a drug reservoir and rate-limiting membrane for releasing the drug [144,145]. The zotarolimus sustained-release stent was adjusted so that 94% of zotarolimus eluted from the MPC polymer layer in 4 weeks [144]. The incidence of stent thrombosis between 2009 and 2020 was as low as 0.28%. It is shown that the suppression of early thrombus formation plays a role in the effectiveness of the MPC polymer layer, minimal invasiveness to intravascular tissues, and drug sustained-release rate control properties.

As for cardiovascular medical devices, artificial hearts have been primarily developed in the United States since the 1970s. Initially, artificial hearts aimed at the same pulsation as a living heart were studied, but the durability of the required elastic material was problematic. Segmented polyurethane (SPU) was developed as a potential material [146]. resulting in a fully transplantable artificial heart in the late 1980s [147]. However, blood compatibility problems and mechanical properties have not been resolved; therefore, it is only widely applied clinically as an auxiliary blood pump outside the body. Studies have also been conducted on surface modifications with zwitterionic polymers to improve blood compatibility [148–152].

However, research on centrifugal pumps that push blood with a propeller rotating under a magnetic field is progressing and these pumps may replace pulsatile flow pumps. With this mechanism, metals can be used for housing and rotating propeller parts to solve mechanical problems. In addition, considering the rotating propeller’s shape, it is relatively easy to control the blood flow; moreover, the system has the advantage of not requiring a valve.

An implantable ventricular assistive heart having a titanium centrifugal pump surface treated with an MPC polymer (PMB) has been developed (EVAHEART; Sun Medical Technology Research Co., Japan) [153]. Safety and operational stability were examined in initial large animal studies. The MPC polymer coating layer remained stable after 180 days of implantation. It was operated for more than 800 days without anticoagulation therapy, and there were no significant changes in blood flow or composition. Clinical trials began in 2005, and implantation procedures were performed in patients with dilated cardiomyopathy [154]. This process, combined with anticoagulation therapy using drugs, has shown significant improvement in cardiac function, enabling patients to attain normalcy rapidly. In 2010, the artificial heart described here became the first implantable artificial heart in Japan for clinical use and more than 230 implantation surgeries have been performed since. Observations of artificial hearts removed from patients undergoing heart transplantation approximately three years after implantation showed that the MPC polymer treatment kept the inner surface of the pump clean and prevented the formation of blood clots. The MPC polymer used was molecularly designed to coat the inner surface of the pump and the SPU cannula that connects to the blood vessels through solvent evaporation [153]. PMB has a hydrophobic BMA unit with a relatively low glass transition temperature; hence, a stable thin film is formed on different substrates and the polymer is stable in the biological environment.

Furthermore, the molecular weight was set to 500 kDa or higher to prevent dissolution in aqueous media, including blood plasma. However, PMB dissolves in alcohol and is easily coated on the substrate using solvent evaporation [48]. It is also secure and registered with the Master Access File of the Food Drug Administration (FDA), U.S.A (MAF2103; LIPIDURE-CM5206).

#### 4.1.2. Cerebrovascular device

Cerebral aneurysms can lead to a subarachnoid hemorrhage, which is a life-threatening condition. Depending on the size of the aneurysm, treatment methods include clipping the aneurysm directly by craniotomy or inserting a metal coil through a catheter to form a thrombus inside the aneurysm, thereby lowering arterial pressure and preventing rupture. However, clipping via craniotomy is a highly invasive procedure and treatment with coil embolization has complications, such as a low healing rate in approximately 20% of cases [155].

A new treatment device, the flow diverter system which applies stent technology, has been developed. This involves the insertion and implantation of a metal stent in the affected area. It significantly reduces blood flow into the cerebral aneurysm while maintaining blood flow in the artery and promoting thrombus formation of the blood accumulated inside the aneurysm to occlude the aneurysm [156]. In 2015, flow-diverting devices were approved in Japan (Pipeline Flex Embolization Device, Medtronic), and several devices were introduced and established as a standard treatment method. To facilitate the insertion of this flow diverter device and reduce initial thrombus formation, the surface of the device was treated with an MPC polymer (Pipeline with Shield Technology^TM^, Medtronic) [157,158]. The MPC polymer (poly(MPC-co-DMA-co-HPMA-co-TMSPMA) (Figure 8(a)) contains monomer units with silane coupling groups and hydroxyl groups that react with the metal strands and form a stable coating layer. The thickness of the surface coating layer is reported to be 3 nm, suggesting that it is approximately a monolayer. *In vitro* and *ex vivo* experiments have shown that the device has good antithrombotic properties, and *in vivo* experiments have also confirmed that the surface is covered with endothelial tissue [159–161]. Representative in vitro blood circulating circuit model results are shown in Figures 8(d,e) [141]. Severe thrombus formation was not observed in the MPC polymer-treated stents. In addition, there was decreased production of the thrombin – antithrombin complex (TAT) in the blood sample after 60 min of contact with the MPC polymer-treated stent (Figure 8(f)). Flow diverter devices generally require a certain period of antiplatelet administration until endothelial tissue covers the surface.

#### 4.1.3. Blood purification devices

Polymer biomaterials are essential in blood separation technology that extracts useful components. Hemodialysis is a life-prolonging treatment for renal failure. Recently, higher-quality blood purification therapy has been introduced that combines the separation processes of dialysis and filtration [162]. In the 1970s, the main materials used were cellulosic materials such as natural polymers. Later, the removal of low molecular weight proteins, such as β2-microglobulin in addition to low molecular weight substances such as creatinine, from the blood was considered important to reduce the incidence of carpal tunnel syndrome caused by long-term hemodialysis treatment. There has been a shift in separation materials to polysulfone-based materials that can easily produce solute-permeable porous membranes [163]. Since polysulfone is a hydrophobic polymer, a hydrophilic polymer is blended with polysulfone to improve the hydrophilicity of the hollow fiber surface when manufacturing hollow fibers used in hemodialysis. Poly(*N*-vinylpyrrolidone) (PVPy) is a commonly used as the hydrophilic polymer [164,165]. Iwasaki *et al*. investigated the use of water-soluble MPC polymers to simultaneously improve the hydrophilicity and hemocompatibility of polysulfone hollow fiber membranes [166]. In the manufacturing of hollow fibers, a double-tube nozzle is used to extrude a polymer solution into the coagulation layer. The coagulation solution inside the nozzle is an aqueous MPC polymer solution that promotes the physical adherence of MPC polymer chains to the inner surface of the produced hollow fiber. When the MPC polymer-treated polysulfone hollow fiber created by this method was passed through the blood as a mini-module, no thrombus formation occurred, even in an anticoagulant-free environment. In contrast, polysulfone and PVPy-treated polysulfone hollow fiber modules clotted, particularly polysulfone hollow fibers, completely occluding the hollow fiber with thrombus. It was simultaneously observed that low-molecular-weight protein permeation could be maintained at a high level. This is attributed to the effective suppression of the protein adsorption layer formation on the surface of the hollow fiber in contact with blood.

Regarding the surface modification of blood purification membranes, there have been many studies on the application of zwitterionic polymers [167,168]. In addition to polysulfone, sulfobetaine-based polymers have been applied to cellulose acetate and poly(vinylidene fluoride) as base polymer materials to improve the hemocompatibility [169–172]. These, as well as MPC polymer-treated polysulfone hollow fibers, have shown good performance. To further develop wearable blood purification devices, research has been conducted on bio-hybrid artificial kidneys, in which kidney cell layers and vascular endothelial cell layers are constructed on the membrane surface [173]. However, the application of new membrane materials in medical devices used for blood purification therapy is currently pending because of the medical economic perspective and the high cost of their quality control during manufacturing.

**Figure 9.**
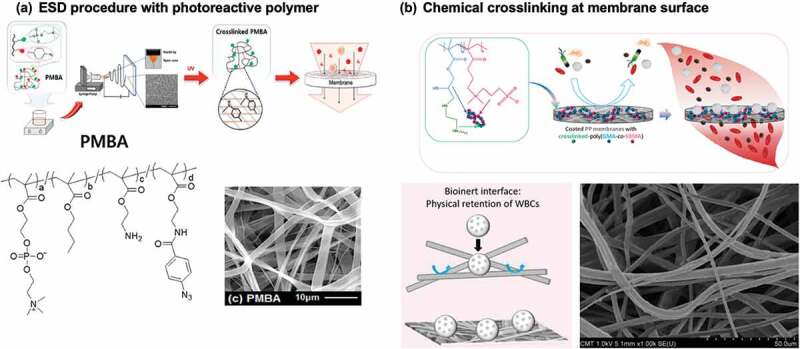
(a) Procedure of MPC polymer (PMBA) nanofiber fabrication by electrospinning deposition (ESD) method and following photoirradiation. Chemical structure of photoreactive PMBA and SEM image of nanofiber membrane are indicated. Reprinted with permission from [174]. Copyright (2017) American chemical society. (b) Surface treatment procedure on polypropylene non-woven membrane with chemically cross-linkable sulfobetaine polymer, poly(gma-*co*-SBMA). Reprinted with permission from [175]. Copyright (2019) elsevier.

In contrast, a large market is expected for blood separation filters to collect stem cells from cord blood or remove immune cells from blood. This requires fine filter components that match the cell size. As a technique for producing nanofibers, the electrospinning deposition (ESD) method, which involves spinning a polymer solution in a high electric field, is used in many polymer systems. The polymers generally applied are relatively hydrophobic. Therefore, to use them as filters for separating biomolecules and cells, it is required to make the fiber surface hydrophilic, perform antifouling treatments, and induce it to interact appropriately with cells. Seo et al. studied the cross-linking and solubilization of a polymer by UV irradiation after producing nanofibers by the ESD method using a photoreactive amphoteric MPC polymer, (Figure 9(a)) [174]. This enables creation of a nanofiber filter that controls mesh density over a large area.

Leukocyte immunity can be a significant obstacle when transfusing blood. In particular, when blood is donated from an unspecified number of people, stored, and used for transfusion, it is necessary to remove leukocytes. Non-woven filters have been developed as leukocyte removal filters, which must be able to pass platelets and red blood cells while capturing leukocytes at a high processing speed. Leukocytes tend to adhere to cationic polymers. A polymer system was thus developed that enables the surface coating of inert nonwoven polypropylene fabrics (Figure 9(b)) [175]. It involves a sulfobetaine polymer with epoxy groups, poly(SBMA-*co*-glycidyl methacrylate (GMA)). When mixed with ethylenediamine in an aqueous solution of the polymer and cast on non-woven fabric, the polymer is cross-linked through the reaction between the epoxy group in the GMA unit and amino groups at 60°C, making it insoluble. A homogeneous polymer coating can then be obtained on the fine non-woven fabric fibers. No substrate pretreatment was required for the reaction, and the reaction process was simple due to the continuous heat immobilization reaction. The separation of blood cell components depends on the size of the blood cells, and leukocytes are removed without passing them through a filter. It is also believed that the amino groups generated by the reaction contribute to the separation efficiency. This process was incorporated into the manufacture of leukocyte removal filters by PuriBlood Co. Compared with conventional filters, these filters has improved platelet and leukocyte removal rates, while the time required for blood recovery is considerably reduced.

#### 4.1.4. Oxygen enrichment device for extracorporeal blood circulation

When living organisms cannot inhale sufficient oxygen due to respiratory failure or when the heart is temporarily stopped due to cardiovascular surgery, it is necessary to artificially supply oxygen and remove carbon dioxide from the blood using an extracorporeal circulation circuit. In this case, a membrane material that combines gas-exchange capability and hemocompatibility is required. The extracorporeal membrane oxygenation (ECMO) treatment has been extremely effective in the treatment of COVID-19 and has saved many patients’ lives [176]. A thin layer of PDMS is formed on a porous hollow fiber obtained from a polyolefin or poly(vinylidene fluoride) base to increase the permeability of oxygen gas while preventing the leakage of water from the plasma [177]. Efficient modification of the PDMS surface with hydrophilic polymers inhibits protein adsorption suppression and enables the maintenance of high oxygen permeability over a long period [178].

**Figure 10.**
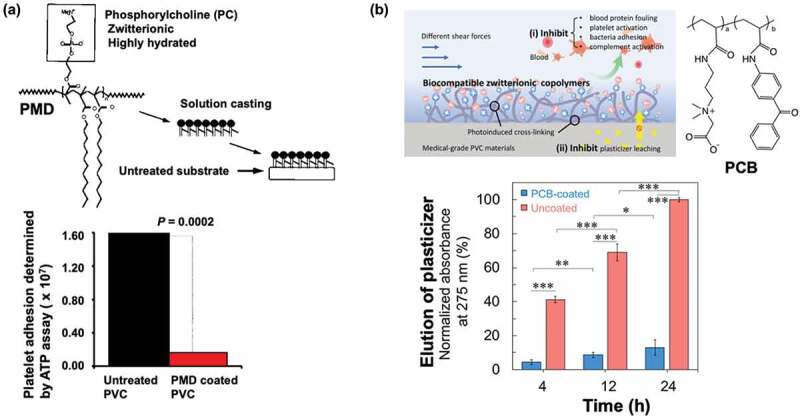
Surface treatment on blood circuit devices. (a) Coating procedure and platelet adhesion examined on PVC treated with MPC polymer (PMD). Reprinted with permission from [181]. Copyright (1994) Walters Kluwer. (b) Photoreaction of carboxybetaine polymer with photoreactive benzophenone group (PCB) on plasticized PVC and reduction of elution of plasticizer by PCB treatment. Reprinted with permission from [135]. Copyright (2020) American chemical society.

Heparin acts effectively on the blood coagulation system and plays a role in preventing blood clotting. Furthermore, it can be expected to reduce the effect on patients by inhibiting the reduction in cell function and activation reactions when blood encounters it. Generally, membrane oxygen-enriched devices surface-treated with heparin are used to prevent thrombus [179]. A substantial amount of heparin is administered to the blood during extracorporeal blood circulation therapy, and coagulation system activity is inhibited in the blood. However, platelet-related coagulation reactions occur on the surface of the membrane material; thus, adhesion and activation of blood cells must be prevented. Although heparinized surfaces are expected to decrease the number of blood cell adhesions due to increased hydrophilicity, they do not prevent platelet adhesion or activation [180]. Therefore, the efficacy of MPC polymers that actively inhibit platelet-based coagulation has been investigated, and poly(MPC-*co*-DMA) (PMD) was applied to the surface treatment of membrane oxygen-enriched devices (Figure 10(a)) [127,179–183]. The Sorin Group was the first to clinically develop it and the device has been shown to inhibit platelet coagulation on the membrane surface and maintain good blood quality in patients after treatment. Subsequently, JMS Co., Japan, also put it into practical use in Japan.

The surface of a blood circuit is an important aspect of extracorporeal therapy. Membrane-type devices have a large surface area and are more prone to reactions with blood on blood-contacting surfaces. In addition to the responses at the contact surfaces of the blood circuit, reactions with blood cells and their components must also be considered. Typically, blood circuits consist of plasticized (vinyl chloride) (PVC) tubing and polycarbonate joints. In extracorporeal therapy, the blood is anticoagulated to prevent thrombus formation during the treatment period.

However, plasticizers in the PVC compound used in the blood circuit may leach out. In an aqueous environment, this effect is negligible. In contrast, the solubilization of the plasticizer due to the high lipophilic substances in the blood is problematic [184]. The application of MPC and carboxybetaine polymers have been studied to solve this problem. The first example is the application of amphiphilic water-insoluble PMD for coating medical materials including PVC and polycarbonate (Figure 10(a)). Fibrinogen adsorption and platelet adhesion can be efficiently suppressed by MPC polymer coating. However, the reduction in plasticizer from PVC is not sufficient after coating with PMD [185]. This may be due to the amphiphilic nature of PMD, as the swelling of the polymer layer maintains the plasticizer’s permeability. Another example is the application of a photoinduced cross-linkable carboxybetaine polymer containing benzophenone groups (PCB) (Figure 10(b)). The polymer is coated onto the surface, and a UV irradiation-induced cross-linking reaction is performed on the plasticized PVC substrate. This has a more pronounced inhibitory effect on plasticizer elution than untreated PVC [135]. These results indicate that the highly hydrophilic carboxybetaine polymer prevents solubilization of the plasticizer from PVC to the polymer layer. The crosslinking of the polymer chain effectively inhibits the diffusion of the plasticizer and stabilizes the substrate’s polymer layer. As the duration of ECMO treatment increases, this technology is expected to become increasingly critical.

### 4.2. Orthopedic devices

Orthopedic surgery has repeatedly utilized artificial materials to replace biological tissues, whose functions have been degraded. Metal medical devices, such as bone fixation plates and screws, have been used to immobilize the affected part and assist in the functional restoration of bone after fractures [186]. Artificial joints are also important medical devices that can dramatically improve patients’ mobile functions and are already being used to improve the function of artificial hip and knee joints, shoulders, and fingers. They are composed of metal parts for fixing to the bone, and polymer parts to form sliding surfaces, taking advantage of their material properties. As joint replacement surgery immediately improves a patient’s mobility, thousands of joint replacement surgeries are performed each year. Stainless steel and cobalt – chromium alloys, which have a high affinity for natural bone, have been used for metal parts. Recently, titanium alloys and materials sprayed with hydroxyapatite, a bone component, have been used to increase bone affinity [187]. Although there have been significant advances in materials for bone immobilization, the effective treatment period for artificial joint replacement is estimated to be approximately 10 years. Osteolysis and resorption around the bone tissue can immobilize the joint prosthesis, resulting in loss of mechanical fixation or loosening [188].

The cause of loosening has been debated for many years. In the 1990s, advances in molecular bone metabolism indicated that it was caused by the materials used [189]. In other words, a ‘particle disease’ is caused by the body’s reaction to submicron-sized wear particles generated around the artificial hip joint. These particles are generated from the sliding surfaces of the joint as it moves during walking and other joint movements. These particles are recognized as foreign substances by macrophages and then phagocytosed. Even small amounts of these particles are subjected to a signal amplification mechanism that is unique to biological reactions. Macrophages secrete cytokines such as tumor necrosis factor (TNF-α), interleukin-6 (IL-6), and prostaglandin E2 (PGE2). These bioactive substances act on the surrounding mesenchymal cells and induce the expression of receptor activator of NF-κB ligand (RANKL), an osteoclast differentiation factor. Consequently, the formation and activity of osteoclasts that absorb (dissolve) bone are promoted, resulting in osteolysis around the artificial hip joint and subsequent laxity of the artificial hip joint [190]. This implies that the material at the joint sliding surface is the cause of the loosening. Until now, polyethylene (PE) has been used for sliding surfaces of artificial joints because of its mechanical properties and inertness under biological conditions [191]. Therefore, a cross-linking reaction is applied to PE liners to increase material strength and reduce wear. However, this causes significant failure, resulting in liner chipping and cracking. In contrast, when ultra-high molecular weight polyethylene (UHMWPE) is used as a liner material, it is found that wear is reduced; cross-linked UHMWPE with mild cross-linking of PE also shows good performance [192]. Highly cross-linked UHMWPE liners irradiated with γ-rays or electron beams of 50 to 100 kGy have been in practical use since the late 1990s and in Japan since 1999, and good clinical results 10 to 15 years after surgery have been reported. According to a comparison of annual linear deformation in a review of these reports, the wear was reduced by 65% with cross-linked UHMWPE (0.042 mm/year compared to 0.137 mm/year with conventional non-cross-linked UHMWPE) [193], although the problem of wear powder inducing bone dissolution has not been resolved.

**Figure 11.**
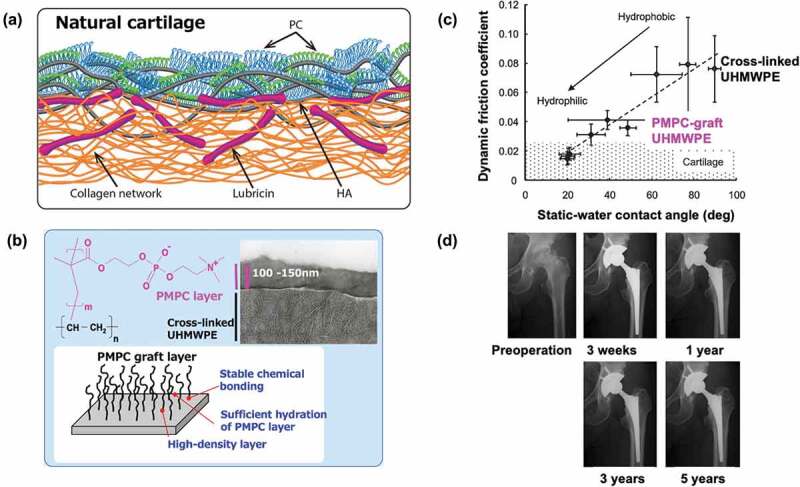
(a) Schematic representation of the surface structure of natural cartilage. Reprinted with permission from [194]. Copyright (2021) Wiley. (b) Photoinduced grafting of PMPC on cross-linked UHMWPE and transmission electron microscope image at the grafting surface. (c) Relationship between static water-contact angle and dynamic friction coefficient of PMPC grafted cross-linked UHMWPE surface. (b) and (c) are reprinted with permission from [195]. Copyright (2008) Wiley. (d) X-ray photo images of PMPC-grafted cross-linked UHMWPE liner installed artificial hip joint implanted into the patient during various implant periods. Reprinted with permission from [196]. Copyright (2017) Wiley.

In biological joints, the cartilage on sliding surfaces is known to play an important role in smooth movement. Anatomical studies have shown that on this surface, hydrophilic proteins and polysaccharides (proteoglycans) form a complex with an additional phospholipid molecular membrane covering the top surface (Figure 11(a)) [194,197]. This structure fixes the joint fluid at the joint interface to avoid direct and indirect contact and allows for easy movement of the joint fluid when a load is applied, followed by restoration of the joint fluid membrane when the load is removed. It is assumed that this fluid lubrication mechanism can be applied to joint prosthesis sliding surfaces. In such cases, wear can be suppressed and osteolysis caused by wear debris and loosening of the joint prosthesis can be prevented. Several studies have shown that surfaces grafted with zwitterionic polymers have better hydrophilicity and lubrication properties [198–203]. However, there are reports that even highly water-absorbent polymers can increase the coefficient of friction [204]. The use of polyelectrolytes, which are expected to act electrostatically, can contribute to an increase in the coefficient of friction.

A method of photoinduced graft polymerization of MPC from the surface of polymer substrates has been devised, and surface modifications were applied to PE [205], UHMWPE [195], PDMS [206,207], and poly(*p*-xylene) (parylene) [208]. In the cases of UHMWPE, a PMPC graft layer can be efficiently formed on the surface by pre-applying benzophenone, immersing the substrate in an aqueous monomer solution, and irradiating it with ultraviolet light (Figure 11(b)) [195]. The graft layer was 100–150 nm. The density of the polymer chains and thickness of the polymer graft layer depended on the photoirradiation time.

Moro *et al*. applied this method to the surface of a cross-linked UHMWPE liner and developed an artificial hip joint liner with excellent surface hydrophilicity and lubrication properties [122]. However, physical-sorption or chemical bonding of MPC polymers on the surface did not significantly improve the lubrication properties against continuous loading [117]. This suggests that the presence of a high density of movable polymer graft chains near the interface plays an important role in the interfacial structure that controls water mobility and that the molecular structure of the PMPC chain is relatively rigid due to the bulky phosphorylcholine groups in the side chains and hydrophilic so that in water, the PMPC chains stand up on the surface because of their hydrophilic nature. In other words, the PMPC graft layer on the surface cooperatively forms a hydration layer with water molecules, creating a good lubrication mechanism (surface hydration gel lubrication mechanism) on the joint sliding surfaces [195]. As shown in Figure 11(c), the dynamic friction coefficient on the surface was linearly related to the water contact angle. When the static water contact angle was approximately 20°, due to the sufficient density of the grafted PMPC chains, the dynamic friction coefficient decreased below 0.02. This value is approximately 1/7 of that of untreated UHMWPE and is minimal compared to the coefficient of friction observed on biological cartilage surfaces [195].

Osteolysis is known to depend on the number of submicron-sized wear particles that macrophages can phagocytose [209]. Long-term sliding tests in a simulation tester simulating human walking motion (in the presence of bovine serum and under a load of 280 kgf) have shown that after 20 million sliding cycles, PMPC-treated cross-linked UHMWPE liners still suppressed the number of wear particles to less than 1% compared to that of the cross-linked UHMWPE [210]. This means that PMPC-treated liners have a very low potential for sagging due to osteolysis. The effect of wear particles produced from PMPC-treated cross-linked UHMWPE liners on bone tissue was examined using macrophages cultured using PMPC-grafted polyethylene particles (0.5 μm, which is the average particle size of the wear powder). In the cell experiments, no production of cytokines or prostaglandins involved in the osteolysis process was observed. These findings can be understood to be the result of a new interface creation technology that simultaneously prevents material wear and significant biological reactions triggered by wear on the sliding surfaces of joint prostheses, thereby extending the life of joint replacement surgery.

This surface technology was implemented in joint prostheses as Aquala Technology by Kyocera Co. in 2011. By June 2022, it has been applied to over 80,000 patients in Japan. Figure 11(d) shows an example of the implantation of an artificial hip joint using the PMPC-treated cross-linked UHMWPE liner [196]. Before the surgery, the patient’s hip joint had limited mobility. After implantation of the artificial hip joint, the joint performance recovered well, and there were no significant problems surrounding the implant for 5 years. The functional improvement index (Japan Orthopedic Association Score) has increased from approximately 43 before surgery to more than 91 after one year, and this value was maintained after 10 years. A single osteolysis case occurred 13–15 years after surgery on the sliding surface. The annual linear deformation is also approximately 0.002 mm/year, which is significantly smaller than that of conventional cross-linked UHMWPE liners, which is an average of 0.042 mm/year, as reported by Kurz *et al* [193].

Surfaces grafted with zwitterionic polymers with good hydrophilicity exhibited properties similar to the surface structure of biological cartilage. Furthermore, it has been reported that the state of the phospholipid layer on the surface of natural cartilage changes with disease, suggesting that the PMPC graft layer, which provides stable phospholipid polar groups, plays an important role [211]. The development of Aquala technology can also offer an advantage when using large-diameter heads with a lower risk of dislocation. This surface modification technology can be used for vitamin E-incorporated liners, which have been applied to reduce functional degradation owing to the oxidation of UHMWPE [212]. The increased longevity of hip arthroplasty is also expected to pave the way for its application to younger patients and bring about significant benefits, such as improved patient quality of life.

**Figure 12.**
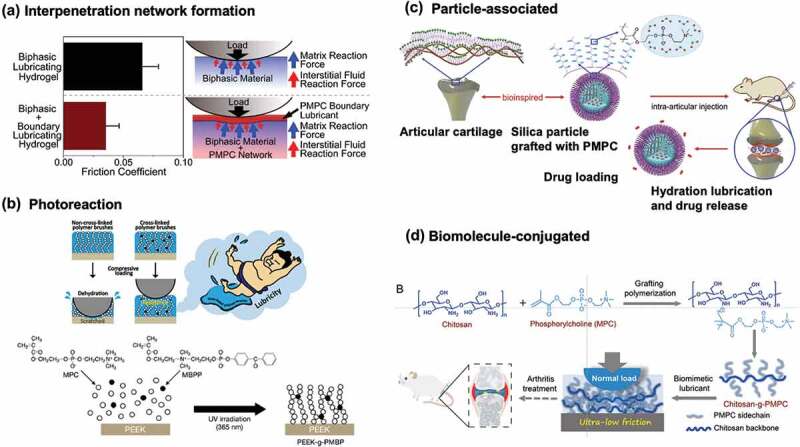
Various stable lubricious surfaces using zwitterionic polymers. (a) PMPC interpenetrating network system. Reprinted with permission from [198]. Copyright (2018) elsevier. (b) Photoreactive cross-linking system to enhance the stability of lubrication layer. Reprinted with permission from [213]. Copyright (2020) American chemical society. (c) a particle-based system using silica nanoparticles covered with MPC grafting layer as lubricant and drug reservoir. Reprinted with permission from [214]. Copyright (2020) elsevier. (d) Bioconjugate system composed of natural biomolecule chitosan and PMPC grafting. Reprinted with permission from [215]. Copyright (2022) Wiley.

Recently, zwitterionic polymers were investigated for the preparation of lubricating surfaces on substrates [198,201,213–218]. They successfully controlled the state of the polymer layer at the interface, thus making it multifunctional. For example, in Figure 12(a), an interpenetrating network structure is formed, which is considered one of the most promising ways to generate a robust hydrogel and improve the load-carrying properties of the sliding interface [198]. Research has been conducted to enhance the durability of polymer brushes by introducing photoreactivity into the polymer brush layer, which achieves high lubrication through a hydration lubrication mechanism and by bridging the polymer brushes (Figure 12(b)) [213]. Silica nanoparticles covered with a zwitterionic polymer brush layer can act as a lubricant that stores drugs to decrease inflammation in joints (Figure 12(c)) [214]. As shown in Figure 12(d), this study mimicked the conditions at the joint interface, focusing on polysaccharide molecules present in the joints and utilizing hybrids with zwitterionic polymers [215]. The aforementioned studies have created materials based on a detailed understanding of the biological properties of joints and the low-friction properties they exhibit and used biomimetic concepts to fabricate these materials. A high-density polymer brush layer was prepared by fixing the zwitterionic polymer to the surface using terminal functional groups or applying surface-initiated polymerization. Suitable solvents expand the polymer chains and the surface achieves fluid lubrication. From these studies, it is expected that new technologies that contribute to mechanical engineering, such as the improvement of energy efficiency owing to low friction and durability by reducing material wear, will be created regardless of medical devices.

### 4.3. Ophthalmology devices

The application of medical devices in the field of ophthalmology began relatively early [38,219]. Spectacles to assist visual function, contact lenses, intraocular lenses to replace lenses, and intraocular drains to regulate intraocular pressure are several examples. Among these, intraocular and contact lenses, which require light transmission and lens function, have been manufactured based on poly(methyl methacrylate) (PMMA) and applied clinically. Intraocular lenses are effective in treating cataracts, and PMMA-based lenses are placed in the eye using polypropylene wire. Several methods for surface modification of MPC polymers have been reported to prevent the progression of corneal cells during this process [220]. In addition, to minimize surgical invasion at the time of placement, a soft acrylic lens was folded and inserted with an applicator, which has become the mainstream method. Recently, intraocular contact lenses made of a collagen and poly(2-hydroxyethyl methacrylate (HEMA)) (PHEMA) composite that does not require lens removal have been clinically used [38].

However, the development of contact lens materials has become increasingly competitive. From the early PMMA-based hard contact lenses, PHEMA-based hydrophilic contact lens materials were developed, considering the wettability on the surface and the suitability of the mechanical properties for soft tissues [221,222]. Generally, cross-linked PHEMA is used as the base material, and water-soluble polymers, such as PAA and PVPy, are combined to balance the water content and mechanical strength factors. In 1995, a cross-linked copolymer composed of HEMA and MPC was developed as a soft contact lens material (Omafilcon A) [131,223]. The FDA has evaluated this lens material as one that can be used for dry eye syndrome because it does not have a beam to the corneal tissue and has good tear fluid circulation.

Contact lenses made of silicone hydrogel-based materials with improved oxygen permeability have been developed [221,224–226]. Many contact lenses can be used continuously from one day to two weeks with ready-for-use pre-packing, increasing user convenience. The oxygen permeability of the lens material is key to continuous wear. When absorbing oxygen from tear fluid, there are two possible methods. First, increasing the diffusion and solubility coefficients. In the case of general hydrogel contact lenses, oxygen dissolution is dependent on dissolution in the water area rather than in the polymer portion [227]. Therefore, the oxygen permeability is a function of the water content of the material.

However, the use of PDMS, which has a higher solubility coefficient of oxygen than water, can be expected to increase the oxygen supply to the eye tissues. Therefore, attempts have been made to introduce PDMS segments into contact lens materials, and many materials have been developed [228,229]. A technique was established to make PDMS domains continuous in the cross-sectional direction to increase solubility. Compositing hydrophilic polymers have improved the wearing comfort, which contributes to the hydrophobic nature of PDMS. In addition, these polymers can improve surface water wettability and lubricity, but oxygen permeability tends to decrease with increasing water content. This correlates the volume fraction of PDMS in the contact lens material with the introduction of hydrophilic polymers and the corresponding increase in the water content.

**Figure 13.**
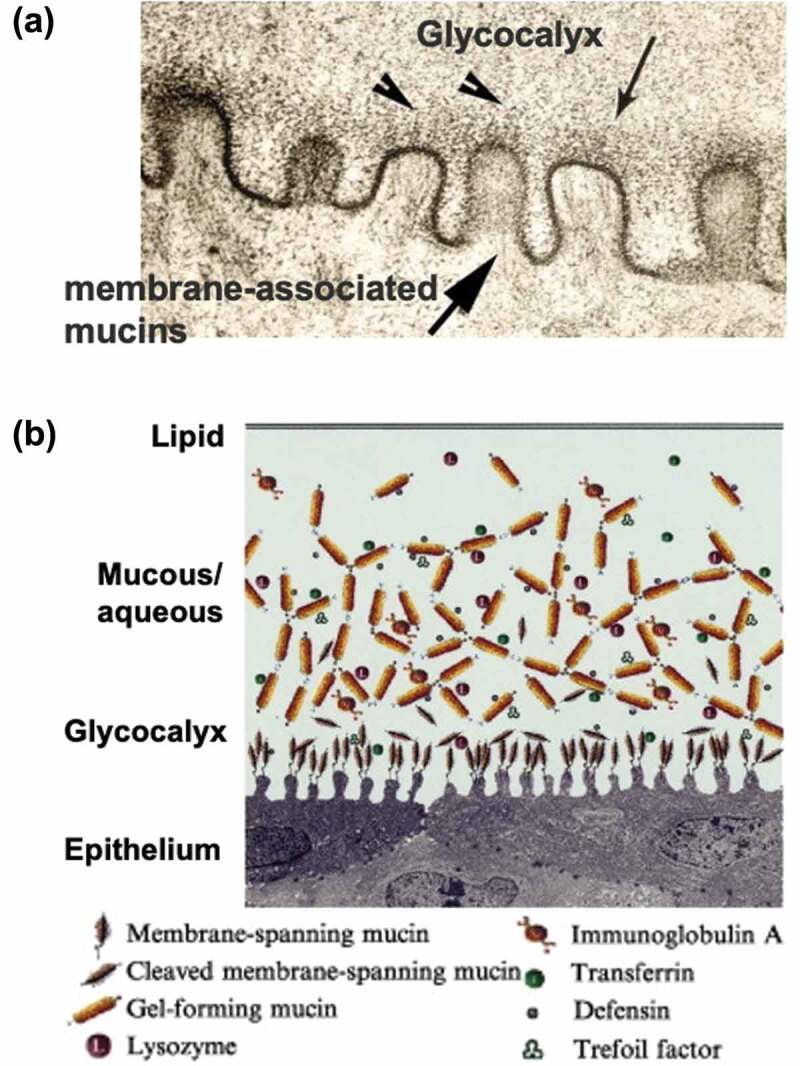
(a) Microstructure of surface of the conjunctiva epithelium observed with a transmission electron microscope. (b) Schematic representation of biomolecules at the surface of the conjunctiva epithelium. Reprinted with permission from [230]. Copyright (2003) elsevier.

Focusing on the structure of the biological corneal surface, the corneal cell surface is covered with bound mucin [230]. Figure 13(a) shows an electron micrograph of the tear film contacting the surface of the conjunctival epithelium. Epithelial cells have a rough endoplasmic reticulum, are synthetically active, and contain small secretory vesicles in the apical cytoplasm. The cell membrane has raised folds, called microplicas, which form regular undulations in the cross-section. These cells express and produce a thick glycocalyx along with membrane-bound mucins, which are important components of the epithelial-lacrimal interface. Figure 13(b) shows a schematic representation of biomolecules at the corneal surface. Free mucin is also present at relatively high concentrations in the tear fluid. These compounds inhibit the evaporation of water from the corneal tissue and prevent dry eye syndrome. If the function of these hydrophilic molecules in a small portion of the surface can be applied to silicone-based contact lens materials, it is thought that both surface properties and oxygen permeability can be solved simultaneously. Although still in the basic stage, research is underway to coat the surface of contact lenses with biomolecules such as mucin and hyaluronic acid to increase lubricity and improve the wearing experience [231,232].s

**Figure 14.**
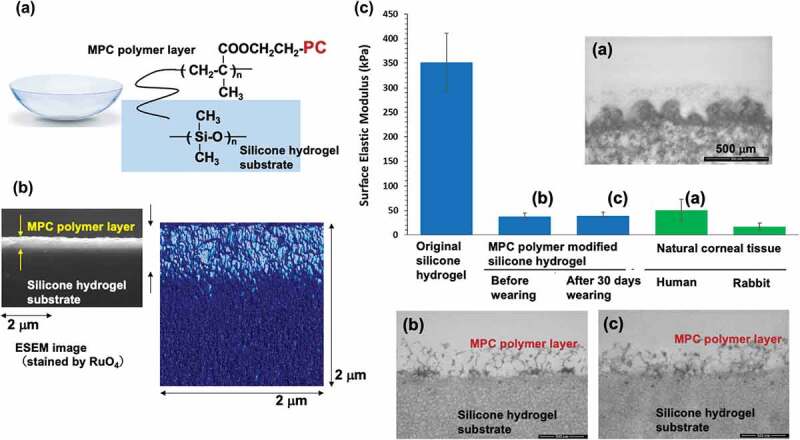
(a) Chemical structure of silicone hydrogel contact lens grafted with the MPC polymer (b) Cross-sectional view of silicone hydrogel contact lens grafted with the MPC polymer. Reprinted with permission from [233]. Copyright (2021) elsevier. (c) Surface elastic properties of silicone hydrogel contact lens and natural corneal tissue. Transmission electron microscopy images (a), (b), and (c) correspond to lettering in the bar graph. Reprinted with permission from [132]. Copyright (2021) American chemical society.

By applying the biomimetic concept to material design, new contact lens materials in which MPC polymers are grafted onto a silicone hydrogel surface have been developed (Lehfilcon A) (Figure 14(a)) [234]. The lens material consisted of a PDMS macromonomer base, a small amount of hydrophilic monomer, and a cross-linking agent. Poly(methacrylic acid) was used to prepare an interpenetrated polymer in a small layer near the base material’s surface, introducing a reactive MPC polymer [233]. In this process, the chemical structure and molecular weight of the MPC polymer are precisely controlled and designed such that the MPC units do not penetrate the substrate. As shown in Figure 14(b), the MPC polymer was concentrated at approximately 200 nm from the surface and phase-separated from the PDMS substrate. There was a water content gradient in the film thickness direction, showing a nonuniform feature. It was observed that surface water wettability was maintained, and the mechanical properties were approximately consistent with those of corneal tissue. The coefficient of kinetic friction was less than 0.05, which is comparable to that of the lubricated surface of biological tissue. The oxygen permeation coefficient was higher than that of conventional silicone hydrogel contact lenses, while the overall water content was shown to be more significant. These characteristics apply to a different area than that of conventional contact lens materials. As shown in Figure 14(c), the MPC polymer treatment on the silicone hydrogel contact lens provided almost the same elastic modulus as that of the natural cornea even when it was worn on the human eye for 30 days continuously [132]. Observation of the microstructure of this contact lens surface using environmental SEM showed that hydrated MPC polymer chains appeared to cover the surface, similar to the surface of natural corneal tissue (Figure 14(c)) [132]. Thus, it is possible to construct a surface with a similar structure and properties by grafting MPC polymers onto the surface of a hydrophobic silicone hydrogel substrate with high mechanical strength. MPC polymer-treated silicone hydrogel material suppresses lipid absorption, protein adsorption, and cell and bacterial adhesion [235]. Therefore, MPC polymer-treated silicone hydrogel materials are expected to be new materials that can be worn continuously for an extended period. These characteristics were attributed to the hydrophilic MPC polymer graft layer on the surface.

## 5. Conclusions and future perspective

Advances in materials science based on biomimetic concepts will contribute significantly to the development of new materials and the creation of new devices. Research and applications of morphological biomimetics and chemical mimetics are advancing at a fast pace. In addition, clarifying the expression of specific functions of living organisms from the viewpoint of molecular structure and reproducing them in artificial systems can be considered one of the major sciences in the creation of functional materials. In biological systems where various molecular reactions occur in a complex manner, there is a strong need to construct biointerfaces that are compatible at the interface of the biological system, rather than using a single material. Polymers that can modify the surface of the base material at the nanometer scale with excellent morphogenetic mechanical properties play an essential role in the development of medical devices. This will not only reduce damage to the biological tissues of in vivo medical devices for an extended period, but will also lead to sustained therapeutic effects and improved quality of life for patients. Furthermore, materials that are compatible with the living body can be considered consistent with the ecosystem and have the potential to provide a means to solve future environmental and energy-related equipment issues. Thus, it is reasonable to apply the biomimetic concept to material design.

This review summarizes the progress of research on biomimetic zwitterionic polymers as biomaterials for their application in medical devices. MPC polymers are the first to be systematically studied and applied to medical devices. The science of MPC polymers has matured, with many researchers conducting fundamental studies. In addition, researches on various zwitterionic polymers are expected to progress further, and new applications in medical devices will be developed. As shown in the several examples discussed in this review, zwitterionic polymers exhibit excellent lubricity and antifouling properties at water interfaces. This may provide important strategies for solving ecological and environmental challenges. For example, possible reduced adhesion of marine organisms [236,237], oil staining prevention [238–240], and the separation of oil from aqueous systems [105,241]. Additionally, particular focus has been placed on the application of zwitterionic polymers as an ion-conducting matrix [242–244]. These material technologies are attractive research targets and can effectively solve future environmental and energy problems.
